# The effect and safety of preoperative biliary drainage in patients with hilar cholangiocarcinoma: an updated meta-analysis

**DOI:** 10.1186/s12957-020-01904-w

**Published:** 2020-07-18

**Authors:** Fei Teng, You-yin Tang, Jun-long Dai, Yongkun Li, Zhe-yu Chen

**Affiliations:** Department of Liver Surgery, Liver Transplantation Center, West China Hospital of Sichuan University, Chengdu, 610041 Sichuan Province China

**Keywords:** Hilar cholangiocarcinoma, Klatskin tumor, Preoperative biliary drainage, Meta-analysis, Total serum bilirubin

## Abstract

**Background:**

The effect and safety of preoperative biliary drainage (PBD) in patients with perihilar cholangiocarcinoma are still controversial; the aim of our study is to evaluate all aspects of PBD.

**Methods:**

All included studies featured PBD versus non-PBD (NPBD) groups were from 1996 to 2019 and were extracted from Cochrane Library, Embase, PubMed, and Science Citation Index Expanded.

**Results:**

Sixteen studies met the inclusion criteria and were included in this analysis. PBD may lead to a significantly higher incidence of overall morbidities (OR 0.67, 95% CI 0.53, 0.85; *P* = 0.0009) and intraoperative transfusions (OR 0.72, 95% CI 0.55, 0.94; *P* = 0.02); moreover, bile leakage (OR 0.58, 95% CI 0.24, 1.41; *P* = 0.04), infection (OR 0.31, 95% CI 0.20, 0.47; *P* < 0.00001), and cholangitis (OR 0.18, 95% CI 0.007, 0.48; *P* = 0.0007) are also related to PBD. However, NPBD was associated with more frequent hepatic insufficiency (OR 3.09, 95% CI 1.15, 8.31; *P* = 0.03). In the subgroup meta-analysis, the differences in the outcomes of bile leakage and overall morbidity lost significance between the PBD and NPBD groups when the mean total serum bilirubin (TSB) concentration was above 15 mg/dl.

**Conclusion:**

Meta-analysis demonstrated that compared to NPBD, PBD is associated with a greater risk of several kinds of infection and morbidities, but its ability to reduce postoperative hepatic insufficiency cannot be ignored. In patients with a high TSB concentration, PBD tends to be a better choice. However, these results need to be confirmed in a future prospective randomized trial with large samples to clarify the effects and find a specific TSB concentration for PBD.

## Introduction

Hilar cholangiocarcinoma (hCCA), which is also known as a Klatskin tumor, is the most prevalent type of all carcinomas related to bile ducts (accounting for 50–60%) [1–3]. hCCA that arises in the proximal extrahepatic epithelium of the bile ducts involving the right and left main hepatic ducts is defined as cholangiocarcinoma. Currently, the only curative treatment for hCCA is surgery that includes extrahepatic bile duct resection, probably with major hepatic resection and potential hepatoduodenal lymphadenectomy [1–4]. However, the majority of patients with hCCA have no typical symptoms until hCCA progresses enough to obstruct the bile ducts and lead to jaundice, which always presents as the first symptom. Unfortunately, according to several studies, patients with jaundice may experience infections, postoperative hepatic insufficiency, intraoperative blood loss, and renal insufficiency [4–8], and preoperative biliary drainage (PBD) is regarded as a practical solution to reduce the total serum bilirubin (TSB) concentration. However, patients who undergo PBD, mainly by percutaneous transhepatic biliary drainage (PTBD) and endoscopic nasobiliary drainage (ENBD), also have an increased risk for tumor seeding, extended hospital stays, morbidities, and infections [1, 2, 9, 10]. Since a balance is needed between benefits and risks, the indications for PBD are still under debate.

In view of this debate, Celotti et al. [11] previously collected nine studies to conduct a meta-analysis of eight kinds of morbidities. However, the number of studies included was small, and several studies that can meet the inclusion criterion have been published in recent years. Moreover, the debate between PBD versus non-PBD (NPBD) for different TSB concentrations also needs to be clarified with a subgroup analysis. Thus, we conducted a systematic review and meta-analysis to fully evaluate the safety and effect of PBD in patients with hCCA.

## Materials and methods

### Literature search

The work has been reported in line with the Preferred Reporting Items for Systematic Reviews and Meta-analyses (PRISMA) and Assessing the Methodological Quality of Systematic Reviews (AMSTAR) guidelines. A systematic literature search was conducted in the Cochrane Library, Embase, PubMed, and Science Citation Index Expanded to identify and retrieve studies published from January 1995 to December 2019 related to the evaluation of PBD in treating hCCA patients. The following concrete search method was used: Search(((PBD[Title/Abstract]) OR drainage[Title/Abstract])) AND (((bile duct cancer[Title/Abstract]) OR *Cholangiocarcinoma*[Title/Abstract]) OR *Klatskin*[Title/Abstract]). The language of the articles was limited in English. The references of each selected study were screened for any relevant articles that could be included.

### Study selection

We set the following inclusion and exclusion criteria for the literature we found. The inclusion criteria were as follows: (1) published in English, (2) was human research, (3) included patients with hilar cholangiocarcinoma, and (4) compared PBD versus NPBD; if the same institution (and authors) published multiple studies, the study with either a higher quality or larger sample size was included. Studies were excluded based on the following criteria: (1) letters, editorials, expert opinions, abstracts, and case reports; and (2) duplicate patient populations.

### Qualitative assessment of the studies selected

The risk of bias in the included non-randomized studies was evaluated according to the risk of bias in non-randomized studies of interventions (ROBINS-1) tool [12].

### Data extraction and synthesis

Each study was screened and evaluated by two investigators independently (Fei Teng and You-yin Tang) for a decision regarding exclusion from the review. If disagreements between the reviewers occurred, a third reviewer (Yongkun Li) was consulted. Two investigators separately collected data from every study included with standardized forms. The patients’ basic characteristics, quality assessments, intraoperative outcomes, and postoperative outcomes were included. The means and standard deviations were used for continuous variable meta-analysis unless otherwise mentioned. If the means and standard deviations were impossible to access with the median, range, and large sample size provided, we used Hozo’s method [13] to approximately estimate the mean and standard deviation.

We extracted the following data from each study: author, year, country, study duration, study design, number of patients in the PBD and NPBD groups, age, sex, body mass index (BMI), TSB before drainage in the PBD groups, preoperative TSB in the NPBD groups, Bismuth classification of the PBD and NPBD groups, mortality, morbidity, hepatic insufficiency, renal insufficiency, R0 resection, operation time (min), need for intraoperative transfusion, and incidence of bile leakage, infection, cholangitis, intra-abdominal abscess, abdominal collection, anastomotic leakage, and second laparotomy.

### Outcomes of interest and definitions

PBD was defined as an approach to reduce serum bilirubin levels before the operation by PTBD or ENBD. The primary outcomes were mortality, which was defined as death occurring within 90 days from admission to the hospital, and morbidity, which was defined as any complication that occurred during hospitalization or within 90 days after surgery. The secondary outcomes included hepatic insufficiency, renal insufficiency, R0 resection, operation time (min), intraoperative transfusion, bile leakage, infection, cholangitis, intra-abdominal abscess, anastomotic leakage, and second laparotomy. Hepatic insufficiency was defined by a standard definition [14]. Renal insufficiency was defined as any decrease in glomerular filtration rate with no reversion to the preoperative level. R0 resection was defined as curative treatment when the resection margin was free of tumor cells according to microscopy. Operation time was defined as the interval from incision to suturing of the skin. Intraoperative transfusion was defined as a blood transfusion of at least 1 unit during the operation. Blood loss was defined as aby blood loss during surgery. Infectious complications were defined according to the study by Hochwald et al. [15] and included infection, intra-abdominal abscess, and cholangitis. Bile leakage was defined as at least 50 ml of bile drained from the surgical drainage tube or from the drainage of an abdominal collection over a period of 3 days or more [16]. Abdominal collection was defined as a collection of fluid in the abdomen after surgery. Anastomotic leakage was defined according to Dindo D’s [17] classification of complications. Second laparotomy was defined as an operation for any curative reason after the previous surgical resection.

### Statistical analysis

A meta-analysis was performed using the Review Manager version 5.3 software (The Cochrane Collaboration, Oxford, UK). For each outcome, *P* < 0.05 was considered statistically significant. Continuous variables are expressed as weighted mean differences (WMDs) and odds ratios (ORs) for comparisons, and their corresponding 95% confidence intervals (CIs) are reported. Categorical variables are reported as ORs with their corresponding 95% confidence intervals (CIs). A chi-square test was used to evaluate heterogeneity, with *P* < 0.1 considered significant. The *I*^2^ value was used to evaluate statistical heterogeneity, and a value of 50% or more indicated the presence of heterogeneity [18]. The fixed-effects model was preferred for all outcomes, but if the test rejected the assumption of homogeneity (*I*^2^ > 50% and heterogeneity *P* > 0.05), we chose the random-effects model. We also performed a sensitivity analysis for every single study included to determine the source of heterogeneity. For every outcome we measured, we performed a funnel plot to evaluate potential publication bias.

## Results

### Description of the included studies

**Fig. 1:**
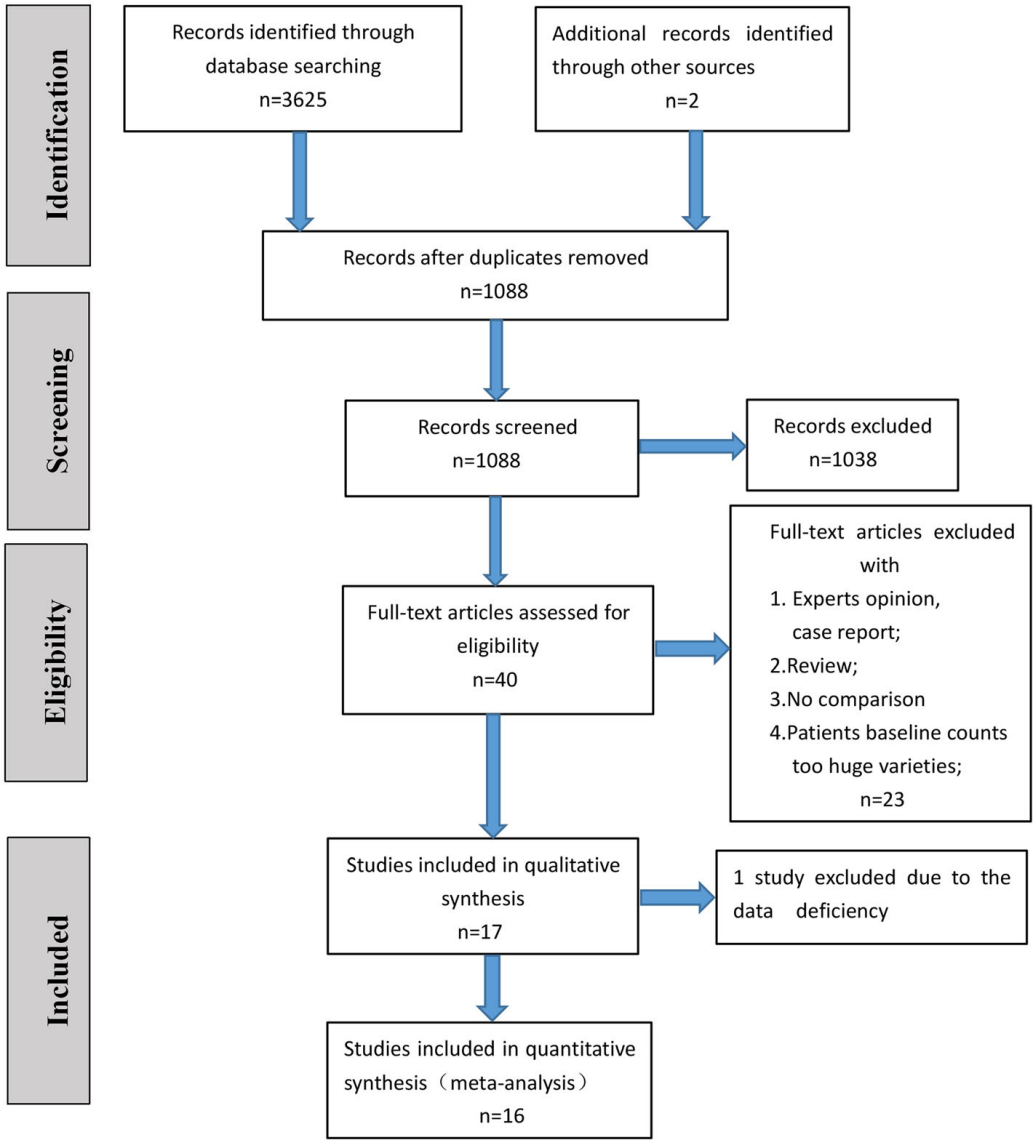
The flow diagram for the searching of studies

**Fig. 2:**
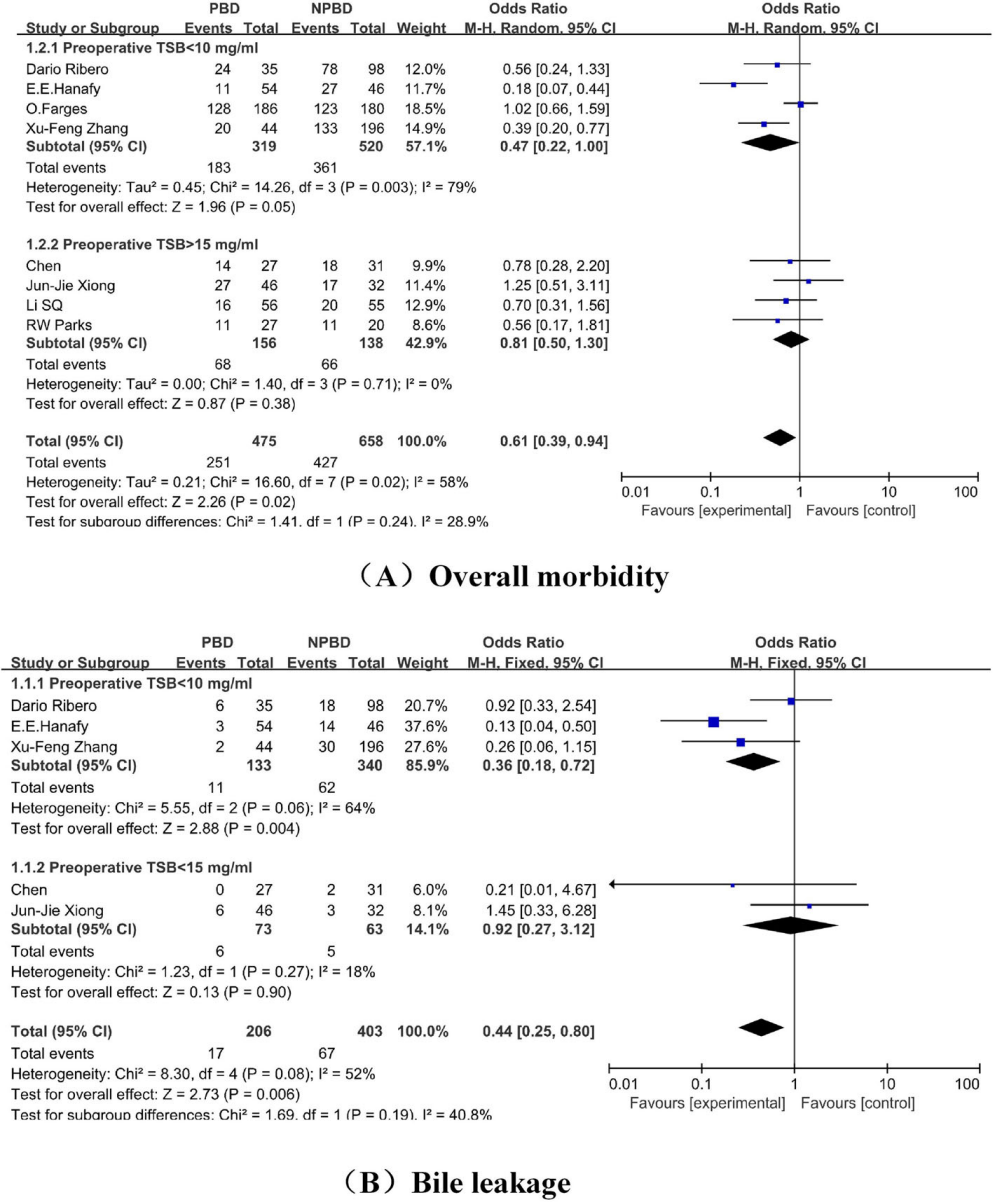
Outcomes of the subgroup meta-analysis (according to the NPBD group’s preoperative TSB concentration in serum). **a** Overall morbidity. **b** Bile leakage

**Fig. 3:**
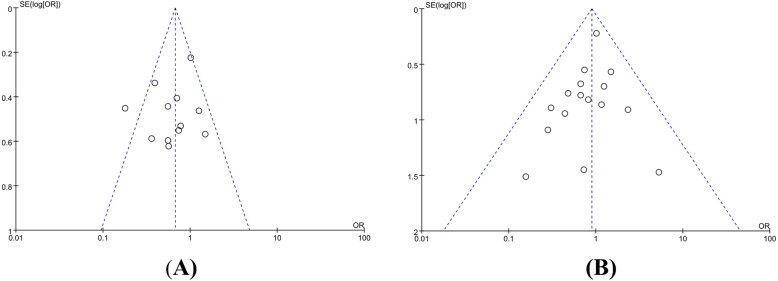
Publication bias measured by the funnel plot of 90-day mortality (**a**) and overall morbidity (**b**)

We reported this systematic review in accordance with the PRISMA statement [19]. A flow diagram of the search process for studies is shown in Fig. 1. In total, 3625 studies were identified from the electronic databases we mentioned, and 2539 studies were removed due to duplicate publications. Finally, only 40 studies were fully screened for eligibility; however, for many reasons, 23 studies were excluded, and only 17 studies were included in our research. Unfortunately, the study conducted by Figueras et al. [20] was excluded after a discussion among our group because of missing data. Thus, 16 studies with 1860 (NPBD, 775 patients; PBD, 1085 patients) patients were included in the meta-analysis [21–36]. Because all of the included studies were retrospective comparative studies, the bias outcomes we measured with the ROBINS-I tool are shown in Table 1. Some studies were considered to have a serious risk of bias [22, 27, 28, 32, 34, 35]; some studies were considered to have a moderate risk of bias [25, 26, 30, 31, 33, 36], and some studies were considered to have a low risk of bias [21, 23, 24]. The characteristics of the included patients are shown in Table 2. The basic perioperative and hilar cholangiocarcinoma data are shown in Tables 3 and 4; the results of the meta-analysis are shown in Table 5; the results of the subgroup meta-analysis are shown in Fig. 2; the publication bias measured by the funnel plot is shown in Fig. 3, and the forest diagrams are updated in supplied materials.

**Table 1:** Risk of bias of studies included.

ROBINS-I	Yulong Cai	Timothy J. Kennedy	XuFeng Zhang	Jun-Jie Xiong	O. Farges	Karolina Maria Wronka	A. Ferrero	C.H.Su	S.Q.Li	Dario Ribero	R.W. parks	Sander Dinant	E.E. Hanafy	G. Ercolani	M.F. Gerhards	Dong Chen
**Bias due to confounding**	L	S	L	L	L	M	S	S	C	L	M	S	L	S	M	L
**Bias in selection of participants into the study**	L	L	L	L	M	L	S	L	L	L	L	L	L	S	L	L
**Bias in measurement of interventions**	L	L	L	L	M	L	L	L	L	L	L	L	L	L	L	L
**Bias due to departures from intended interventions**	L	L	L	L	L	L	L	L	L	L	L	L	L	L	M	L
**Bias due to missing data**	L	S	L	L	M	M	L	L	L	M	M	M	M	L	L	L
**Bias in measurement of outcomes**	L	L	L	L	L	L	L	L	L	L	L	L	L	L	L	L
**Bias in selection of the reported results**	L	M	L	L	L	L	M	S	M	M	L	S	L	M	S	M
**Overall**	L	S	L	L	M	M	S	S	C	M	M	S	M	S	S	M

*ROBINS-*Irisk of bias in non-randomized studies of interventions, *L* low risk, *M* moderate risk, *S* serious risk, *C* critical risk

**Table 2:** Characteristics of the studies included

Author	Country	Year	Design	No of patients		Age		TSB(μmol/l)		Sex (M/F)		BMI		Bismuth-classification	
				NPBD	PBD	NPBD	PBD	NPBD	PBD	NPBD	PND	NPBD	PBD	NPBD	PBD
**Cai et al.** [21]	CN	2017	RETRO	163	55	60 ± 9	59 ± 11	161.0 (8.4–455.6)	281.1 (8.2–565.4)	74/89	30/25	22.5 ± 6.8	21.8 ± 5.9	24/28/24/42/45	9/13/10/8/5
**Kennedy et al.** [22]	USA	2009	RETRO	29	31	NR	NR	NR	NR	NR	NR	NR	NR	NR	NR
**Zhang et al.** [23]	CN	2017	RETRO	44	196	70	67	30.8 (15.4–99.2)	99.2 (39.3–205.2)	20/22	113/83	25.5 (23.7–28.7)	25.0 (22.3–28.7)	15 (I–II)/37 (III–IV)	49 (I–II)/130 (III–IV)
**Xiong et al.** [24]	CN	2013	RETRO	46	32	58.2 ± 11.3	59.6 ± 11.0	254.0 ± 63.5	294.2 ± 135.7	28/18	21/11	21.0 ± 2.5	20.3±1.9	1/14/7/15/9	1/8/6/9/8
**Farges et al.** [25]	EURO	2012	RETRO	186	180	62 ± 11	61 ± 10	126 (17–255)	113 (40–274)	107/79	127/53	24 ± 4	25 ± 4	6/47/267/19/27	4/23/137/10/6
**Wronka et al.** [26]	Poland	2019	RETRO	23	58	56 (48–65)	60 (55–67)	164.8 (18.8–336.4)	36.6 (15.9–83.8)	13/10	34/24	NR	NR	NR	NR
**Ferrero et al.** [27]	Italy	2008	RETRO	30	30	63.5 (35–80)	66.0 (47–80)	210.3 (59.9–533.5)	225.7	19/11	13/17	NR	NR	NR	NR
**Su et al.** [28]	USA	1996	RETRO	16	33	NR	NR	NR	NR	NR	NR	NR	NR	NR	NR
**Li et al.** [29]	CN	2009	RETRO	56	55	NR	NR	268 ± 174	256 ± 136	NR	NR	NR	NR	NR	NR
**Ribero et al.** [30]	Italy	2016	RETRO	35	98	67 (35–82)	65 (40–84)	94.1	63.3	22/13	62/36	NR	NR	0/8/12/13/2	0/17/43/27/11
**Parks et al.** [31]	UK	2000	RETRO	27	20	59 ± 3	57 ± 2	335 (9–670)	278 (35–666)	20/7	16/4	NR	NR	2/7/11/5/2	2/2/10/2/4
**Dinant et al.** [32]	Netherlands	2006	RETRO	14	83	NR	NR	NR	NR	NR	NR	NR	NR	NR	NR
**Hanafy** [33]	Egypt	2010	RETRO	54	46	50.4 ± 12	53.3±11	314.6 ± 205.2	92.34 ± 126.5	34/20	30/16	NR	NR	NR	NR
**Ercolani et al.** [34]	Japan	2010	RETRO	7	44	NR	NR	NR	NR	NR	NR	NR	NR	NR	NR
**Gerhards et al.** [35]	Netherlands	1999	RETRO	18	93	NR	NR	NR	NR	NR	NR	NR	NR	NR	NR
**Chen et al.** [36]	CN	2007	RETRO	27	31	NR	NR	382 ± 174	292 ± 103	NR	NR	NR	NR	2/6/3/7/9	1/4/6/12/8

Data shown represents mean ± standard deviation or median (minimum-maximum); Bismuth-classification = I/II/IIIa/IIIb/

*TSB* total serum bilirubin, *No* number, *M* male, *F* female, *BMI* body mass index, *PBD* preoperative biliary drainage group, *NPBD* non-preoperative biliary drainage group, *Retro* retrospective, *NR* not report

**Table 3:** Operative data of primary outcomes and major complications

Author	Mortality		Morbidity		Hepatic insufficiency		Renal insufficiency		Intraoperative transfusion		Bile leak		R0	
	NPBD	PBD	NPBD	PBD	NPBD	PBD	NPBD	PBD	NPBD	PBD	NPBD	PBD	NPBD	PBD
**Cai et al.** [21]	7	0	NR	NR	NR	NR	NR	NR	57	26	NR	NR	125	42
**Kennedy et al.** [22]	4	2	NR	NR	5	0	NR	NR	NR	NR	NR	NR	NR	NR
**Zhang et al.** [23]	2	13	20	133	NR	NR	NR	NR	13	71	2	30	32	124
**Xiong et al.** [24]	2	3	27	17	6	3	4	3	24	11	6	3	NR	NR
**Farges et al.** [25]	22	17	128	123	14	5	NR	NR	83	97	NR	NR	NR	NR
**Wronka et al.** [26]	1	8	NR	NR	1	1	NR	NR	2	0	NR	NR	NR	NR
**Ferrero et al.** [27]	3	1	19	21	4	5	NR	NR	17	21	10	7	NR	NR
**Su et al.** [28]	0	5	6	17	NR	NR	NR	NR	NR	NR	NR	NR	NR	NR
**Li et al.** [29]	5	4	16	20	NR	NR	NR	NR	NR	NR	NR	NR	37	30
**Ribero et al.** [30]	3	12	24	78	23	6	NR	NR	NR	NR	6	18	32	82
**Parks et al.** [31]	1	1	11	11	NR	NR	1	1	NR	NR	NR	NR	NR	NR
**Dinant et al.** [32]	2	14	6	56	NR	NR	NR	NR	NR	NR	NR	NR	NR	NR
**Hanafy** [33]	3	5	11	27	5	8	0	2	7	15	3	14	NR	NR
**Ercolani et al.** [34]	2	25	NR	NR	NR	NR	NR	NR	NR	NR	NR	NR	NR	NR
**Gerhards et al.** [35]	3	16	13	59	NR	NR	NR	NR	NR	NR	NR	NR	NR	NR
**Chen et al.** [36]	3	3	14	18	4	2	4	1	NR	NR	0	2	11	13

Continuous data shown represents mean ± standard deviation or median (minimum-maximum)

*NR* not report, *PBD* preoperative biliary drainage group, *NPBD* non-preoperative biliary drainage group

**Table 4:** Operative data of overall complications

Author	Infection rate		Cholangitis		Intra-abdominal abscess		Intraoperative blood loss(ml)		Operation time(min)		Second laparotomy		Anastomotic leakage		Abdominal collection	
	NPBD	PBD	NPBD	PBD	NPBD	PBD	NPBD	PBD	NPBD	PBD	NPBD	PBD	NPBD	PBD	NPBD	PBD
**Cai et al.** [21]	NR	NR	NR	NR	NR	NR	1012.5 ± 491.7	325 ± 25	362.5 ± 121.7	370 ± 175	10	1	NR	NR	NR	NR
**Kennedy et al.** [22]	NR	NR	NR	NR	NR	NR	NR	NR	NR	NR	NR	NR	NR	NR	NR	NR
**Zhang et al.** [23]	6	44	NR	NR	4	36	428.25 ± 190.75	509 ± 173	NR	NR	NR	NR	1	10	NR	NR
**Xiong et al.** [24]	11	13	2	1	2	3	675 ± 275	675 ± 225	NR	NR	3	2	2	1	9	6
**Farges et al.** [25]	1	6	NR	NR	NR	NR	NR	NR	330M	385M	NR	NR	NR	NR	NR	NR
**Wronka et al.** [26]	9	17	NR	NR	NR	NR	NR	NR	349.5 ± 124.5	312 ± 117	NR	NR	NR	NR	NR	NR
**Ferrero et al.** [27]	5	12	1	3	2	4	NR	NR	NR	NR	3	4	NR	NR	4	4
**Su et al.** [28]	NR	NR	NR	NR	NR	NR	NR	NR	NR	NR	NR	NR	NR	NR	NR	NR
**Li et al.** [29]	NR	NR	NR	NR	NR	NR	NR	NR	NR	NR	NR	NR	NR	NR	NR	NR
**Ribero et al.** [30]	NR	NR	2	40	NR	NR	825 ± 375.0	1164 ± 583.3	365 ± 147.5	551 ± 216.7	NR	NR	NR	NR	NR	NR
**Parks et al.** [31]	3	13	NR	NR	NR	NR	NR	NR	NR	NR	NR	NR	0	1	NR	NR
**Dinant et al.** [32]	NR	NR	NR	NR	NR	NR	NR	NR	NR	NR	NR	NR	NR	NR	NR	NR
**Hanafy** [33]	11	27	NR	NR	NR	NR	NR	NR	246 ± 96	348 ± 138	NR	NR	NR	NR	NR	NR
**Ercolani et al.** [34]	NR	NR	NR	NR	NR	NR	NR	NR	NR	NR	NR	NR	NR	NR	NR	NR
**Gerhards et al.** [35]	NR	NR	NR	NR	NR	NR	NR	NR	NR	NR	NR	NR	NR	NR	NR	NR
**Chen et al.** [36]	4	6	NR	NR	1	1	848 ± 1112	1016 ± 923	NR	NR	NR	NR	NR	NR	2	4

Continuous data shown represents mean ± standard deviation or median (minimum-maximum)

*NR* not report, *NPBD* non-preoperative biliary drainage group, *PBD* preoperative biliary drainage group

**Table 5:** Results of meta-analysis comparing NPBD versus PBD for hCCA

Outcome of interest	No. of studies	No. of patients	OR/WMD	95% CI	***P*** value	Heterogeneity ***P*** value	***I***^2^
**Primary outcomes**							
**Mortality**	16	775/1085	0.91	0.64, 1.30	0.62	0.78	0
**Morbidity**	12	553/897	0.67	0.53, 0.85	0.0009	0.05	0.44
**Secondary outcomes**							
**Hepatic insufficiency**	8	430/506	3.09	1.15, 8.31	0.03	0.001	0.71
**Renal insufficiency**	4	154/129	1.07	0.40, 2.84	0.89	0.33	0.13
**Intraoperative transfusion**	7	546/597	0.72	0.55, 0.94	0.02	0.07	0.49
**Bile leak**	6	236/433	0.58	1.24, 1.41	0.04	0.04	57%
**R0**	5	325/435	1.36	0.93, 1.96	0.11	0.77	0%
**Infection**	8	414/535	0.31	0.20, 0.47	< 0.001	0.16	16%
**Cholangitis**	3	111/160	0.18	0.007, 0.48	0.0007	0.15	48%
**Intra-abdominal abscess**	5	147/289	0.48	0.22, 1.32	0.07	0.94	0%
**Intraoperative blood loss**	5	315/412	32.34	− 375.83, 440.51	0.88	< 0.001	99%
**Operative time**	4	235/294	− 63.21	− 156.16, 29.73	0.18	< 0.001	90%
**Secondary laparotomy**	4	239/117	1.37	0.52, 3.63	0.53	0.47	0%
**Anastomotic leakage**	3	117/248	0.55	0.15, 2.10	0.38	0.64	0%
**Abdominal collection**	3	103/93	0.9	0.40, 2.00	0.79	0.82	0%

The charts filled with gray means valid outcomes (*P* < 0.05)

*NPBD* non-preoperative biliary drainage, *PBD* preoperative biliary drainage, *No. of patients* NPBD group/PBD group, *OR* odds ratio, *WMD* weighted mean difference, *hCCAAA* hilar cholangiocarcinoma

### Results of the meta-analysis

After a common meta-analysis, to evaluate the effects of PBD among different preoperative TSB levels in patients with hCCA, the included studies were divided into two subgroups: TSB < 10 mg/dl before PBD [21, 23, 25, 26, 30], and TSB > 15 mg/dl before PBD [24, 29, 31, 33, 36]. However, the outcomes presented only statistically significant differences in morbidity and bile leakage between subgroups.

#### Primary outcomes

The mortality rate was reported in all 16 studies [21–36], with no statistically significant difference observed between NPBD and PBD (OR 0.91, 95% CI 0.64, 1.30; *P* = 0.62). The outcome of morbidity was reported in 12 studies [23–25, 27–33, 35, 36], with a significant difference observed between groups; this result showed that PBD may lead to more morbidities (OR 0.67, 95% CI 0.53, 0.85; *P* = 0.0009).

#### Secondary outcomes

Hepatic insufficiency was reported in 8 studies [22, 24–27, 30, 33, 36], and the NPBD group had a significantly higher risk of suffering from hepatic insufficiency than the PBD group (OR 3.09, 95% CI 1.15, 8.31; *P* = 0.03). Renal insufficiency was reported in 4 studies [24, 31, 33, 36], and no significant difference was observed between groups (OR 1.07, 95% CI 0.40, 2.84; *P* = 0.89). Intraoperative transfusion was reported in 7 studies [21, 23–27, 33], and the PBD group had a significantly higher risk of requiring intraoperative transfusions (OR 0.72, 95% CI 0.55, 0.94; *P* = 0.02). The PBD group had a significantly higher incidence of bile leakage according to the outcomes of 6 studies [23, 24, 27, 30, 33, 36] (OR 0.58, 95% CI 0.24, 1.41; *P* = 0.04). The proportion of positive surgical margins was reported in 5 studies [21, 23, 29, 30, 36], and there was no statistically significant difference between groups (OR 1.36, 95% CI 0.93, 1.96; *P* = 0.11).

Infection was significantly more likely to occur in the PBD group than in the NPBD group, based on the outcomes of 8 studies [23–27, 31, 33, 36] (OR 0.31, 95% CI 0.20, 0.47; *P* < 0.00001). Cholangitis was reported in only 3 studies [24, 27, 30], and the PBD group had a significantly higher risk of cholangitis (OR 0.18, 95% CI 0.007, 0.48; *P* = 0.0007). Intra-abdominal abscess was reported in 5 studies [23–25, 27, 36], without a statistically significant difference between groups (OR 0.48, 95% CI 0.22, 1.32; *P* = 0.07). Intraoperative blood loss was reported in 5 studies [21, 23, 24, 30, 36], without a statistically significant difference between groups (WMD 32.34, 95% CI 375.83, 440.51; *P* = 0.88). The operation time was reported in 5 studies [21, 25, 26, 30, 33], without a statistically significant difference between groups (WMD − 63.21, 95% CI − 156.16, 29.73; *P* = 0.18). Second laparotomy was reported in 4 studies [21, 24, 26, 27], without a statistically significant difference between groups (OR 1.37, 95% CI 0.52, 3.63; *P* = 0.53). Anastomotic leakage was only reported in 3 studies [23, 24, 31], without a statistically significant difference between groups (OR 0.55, 95% CI 0.15, 2.10; *P* = 0.38). Abdominal collection was reported in 3 studies [24, 27, 36], without a statistically significant difference between groups (OR 0.9, 95% CI 0.4, 2.00; *P* = 0.79).

#### Subgroup meta-analysis

All studies were divided into low TSB concentrations (< 10 mg/dl) [21, 23, 25, 26, 27] and high TSB concentrations (> 15 mg/dl) [24, 29, 31, 33, 36] according to the mean TSB concentration. All of the primary outcomes and secondary outcomes mentioned were analyzed, but statistically significant differences were only observed in overall morbidity (low TSB concentration: OR = 0.47, 95% CI 0.22, 1.00, *P* = 0.05; high TSB concentration: OR = 0.81, 95% CI 0.50, 1.30, *P* = 0.38) and bile leakage (low TSB concentration: OR = 0.36, 95% CI 0.18, 0.72, *P* = 0.004; high TSB concentration: OR = 0.92, 95% CI 0.27, 3.12, *P* = 0.90).

### Sensitivity analysis

We conducted a sensitivity analysis on every study included by changing the type of effects model or excluding individual studies from the outcomes analysis. No results changed when the effect model was simply switched to another model. Although high heterogeneity existed in the outcomes of intraoperative blood loss, hepatic insufficiency, and operation time, the outcomes were stable, with no meaningful or significant changes when the effect model was changed. There was high heterogeneity for liver insufficiency; however, the heterogeneity was zero when Dario’s study [30] was excluded.

## Discussion

For several years, jaundice after hepatectomy was regarded as an incident related to hepatic insufficiency or even hepatic failure; moreover, the main reason for death after liver surgery was hepatic failure [37]. In fact, the mechanisms of liver failure caused by hyperbilirubinemia have already been demonstrated in animal experiments, and cholestasis makes the liver more susceptible to ischemia, reperfusion drainage, and inflammation, likely because of a reduction in antioxidant ability and a stronger response to inflammation [38]. However, the routine use of PBD in hCCA is still controversial in clinical practice.

Many experts and researchers from Western countries and Japan suggested the routine use of PBD before surgery in hCCA patients [22, 39–41] due to its effects in reducing liver insufficiency. However, the most recent opinion that PBD could not improve the primary outcomes of hCCA patients was suggested by Wronka [26] and Zhang et al. [23], who worried about the complications associated with PBD, including tumor seeding, cholangitis, inflammation, and additional infections. The aim of PBD is to increase liver tolerance to ischemia and reduce intraoperative blood loss [42], and several studies have already demonstrated the significance of PBD [43–45]. However, most of these studies showed benefits without reporting specific characteristics, such as tumor size, concentration of serum bilirubin, liver remnant volume, preoperative complications, and Bismuth classification, so the standard of how and when to use PBD in patients with hCCA is still not clear. In many medical care centers, doctors develop their own suggestion for conducting PBD regarding preoperative TSB concentration: Nimura et al. [46] and Makuuchi et al. [47] suggested PBD at a bilirubin cut-off of 3 mg/dl to minimize the occurrence of complications, and Hemming et al. [37] preferred 5 mg/dl of TSB for PBD. However, Su et al. [28] and Ercolani et al. [34] suggested that when the TSB concentration exceeded 10 mg/dl, the operation should be delayed, and PBD was required. However, whether PBD with the aim of decreasing the TSB concentration surely improves liver tolerance to perioperative inflammation and ischemia, while considering the increased incidence of tumor seeding, infection, cholangitis, etc., and how to balance the benefits and risks are still unclear.

Regarding the primary outcomes, no statistically significant difference in mortality was found between the PBD and NPBD groups; however, the overall morbidity rate was higher in the PBD group. Interestingly, the overall morbidity rate changed when the studies were divided into the low and high TSB concentration groups. In the low TSB concentration group, the OR was 0.47, with a statistically significant difference between PBD and NPBD; the OR in the high concentration group reached 0.81, without a statistically significant difference between PBD and NPBD. At lower TSB concentrations, PBD might increase the risk of infection in the bile duct system [48]; however, as the concentration of TSB increases, liver function is impaired, and the NPBD patient with severe jaundice who underwent surgery may bare more risk for morbidities.

We performed the subgroup analysis based on bilirubin less than 10 mg/ml and greater than 15 mg/ml in the preoperative NPBD group; however, it is undeniable that even in the low concentration group, there may be a very few patients with more than 15 mg/ml of high concentration group with lower preoperative TSB concentration. Therefore, the results of the subgroup analysis are very conservative and can only be used as a reference for future related research directions. The difference between the low and high TSB concentration groups most likely indicates that there was a reasonable concentration of TSB for hCCA patients that motivated the decision to perform PBD.

Regarding the secondary outcomes, no statistically significant difference was observed in renal insufficiency, positive surgical margin, intra-abdominal abscess, intraoperative blood loss, operation time, second laparotomy, anastomotic leakage, or abdominal collection between the two groups. In contrast, statistically significant differences were found in hepatic insufficiency, intraoperative transfusion, bile leakage, infection, and cholangitis.

Interestingly, all of the outcomes with a statistically significant difference were consistent with the fact that the NPBD group might have a lower risk of developing infectious complications and requiring intraoperative transfusions. Stents in the bile duct system surely led to a higher infection rate (cholangitis, infection, intra-abdominal abscess), which may lead to a higher mortality rate, especially when the liver remnant function (FLR) is < 30% [30].

On the other hand, PBD decreased the hepatic insufficiency rate but to the same degree in both the low and high TSB concentration groups; this was likely caused by the approximately same short duration of high TSB poisoning when the different concentrations of TSB increase according to the degree of obstruction. In the subgroup analysis of bile leakage, similar to that of overall morbidity, within the low TSB concentration group, the NPBD group had a significantly lower risk of bile leakage (OR = 0.36), while in the high TSB concentration group, the OR changed to 0.92, and the difference lost significance. On the one hand, this confusing conclusion may be caused by the small number of studies (only two studies) in the high TSB concentration group. On the other hand, PBD had already injured the bile duct physically or by infection; thus, when the concentration of TSB increased, the cicatrization after surgery slowed down, and the liver tended to be more susceptible to inflammation [28, 49].

Currently, several studies have discussed whether PBD can be performed for hCCA patients with different conditions. Wiggers et al. [49] and Kennedy et al. [22] showed that patients with an FLR < 30% who underwent PBD could achieve a significantly reduced hepatic insufficiency rate (33% versus 0%, PBD versus NPBD) and mortality rate (33% versus 0%, PBD versus NPBD); however, in patients with an FLR > 30%, PBD was not superior to NPBD in reducing the hepatic insufficiency rate (0% versus 0%) or mortality rate (0% versus 9.1%, NPBD versus PBD). Wronka et al. [26] claimed that PBD was not necessary if hCCA patients had a TSB concentration > 6.2 mg/dl or > 2.50 mg/dl with preoperative hypoalbuminemia, anemia, or renal dysfunction.

According to our meta-analysis outcomes, PBD was not suitable for patients with hCCA because of the increased risk of bile leakage, cholangitis, infection, intraoperative transfusion, and overall morbidity. However, patients with hCCA and a TSB concentration > 15 mg/dl may still tend to undergo PBD rather than NPBD due to the lower mortality rate of PBD, especially in patients with a low FLR.

Moreover, clinical hCCA patients may suffer other diseases or conditions (hypoalbuminemia, anemia, renal dysfunction, renal failure, low immune function, etc.), and the benefits and risks of PBD should be evaluated separately to meet a standard. Nevertheless, high-quality retrospective studies and randomized controlled trials (RCTs) are required to draw definitive guidelines for hCCA patients with different conditions.

The meta-analysis still has some limitations. First, the subgroups were roughly divided by the mean TSB of each study (< 10 mg/dl and > 15 mg/dl). Although we communicated with the authors of the studies to acquire the primary data and widened the TSB difference between the two groups (< 10 mg/dl and > 15 mg/dl), it was still difficult to obtain fully convincing subgroup outcomes. Second, all of the studies were retrospective studies, which could lead to inevitable selection bias. Third, the studies by Su et al. [28] and Gerhards et al. [35] date back to 1996 and 1999 and perhaps apply different surgical techniques that might influence the results. Fourth, the drainage types included ENBD and PTBD, which were not mentioned in most of the studies included. Therefore, it is difficult for us to evaluate the bias due to the proportion of ENBD and PTBD procedures performed, although no large differences seemed to be observed.

## Conclusion

The meta-analysis demonstrated that PBD is related to a greater risk of several kinds of infections and morbidities than NPBD, but the ability of PBD to reduce postoperative hepatic insufficiency cannot be ignored. In patients with a high TSB concentration, PBD tends to be a better choice. Nonetheless, these results need to be confirmed in a future prospective randomized trial with large samples to clarify the effects and find a specific TSB concentration for PBD.

## Data Availability

The tables and figures supporting the conclusions of this article are included within the article and additional files.

## Abbreviations

CI: Confidence interval
OR: Odds ratio
RR: Relative risk
PBD: Preoperative biliary drainage
NPBP: Non-preoperative biliary drainage
hCCA: Hilar cholangiocarcinoma
TSB: Total serum bilirubin
BMI: Body mass index
FLR: Future liver remnant
PTBD: Percutaneous, transhepatic biliary drainage
ENBD: Endoscopic nasobiliary drainage
